# Review of Generative Adversarial Networks in mono- and cross-modal biomedical image registration

**DOI:** 10.3389/fninf.2022.933230

**Published:** 2022-11-22

**Authors:** Tingting Han, Jun Wu, Wenting Luo, Huiming Wang, Zhe Jin, Lei Qu

**Affiliations:** ^1^Ministry of Education Key Laboratory of Intelligent Computing and Signal Processing, Information Materials and Intelligent Sensing Laboratory of Anhui Province, Anhui University, Hefei, China; ^2^School of Artificial Intelligence, Anhui University, Hefei, China; ^3^Institute of Artificial Intelligence, Hefei Comprehensive National Science Center, Hefei, China; ^4^SEU-ALLEN Joint Center, Institute for Brain and Intelligence, Southeast University, Nanjing, China

**Keywords:** cross-modal, biomedical image registration, Generative Adversarial Networks, image translation, adversarial training

## Abstract

Biomedical image registration refers to aligning corresponding anatomical structures among different images, which is critical to many tasks, such as brain atlas building, tumor growth monitoring, and image fusion-based medical diagnosis. However, high-throughput biomedical image registration remains challenging due to inherent variations in the intensity, texture, and anatomy resulting from different imaging modalities, different sample preparation methods, or different developmental stages of the imaged subject. Recently, Generative Adversarial Networks (GAN) have attracted increasing interest in both mono- and cross-modal biomedical image registrations due to their special ability to eliminate the modal variance and their adversarial training strategy. This paper provides a comprehensive survey of the GAN-based mono- and cross-modal biomedical image registration methods. According to the different implementation strategies, we organize the GAN-based mono- and cross-modal biomedical image registration methods into four categories: modality translation, symmetric learning, adversarial strategies, and joint training. The key concepts, the main contributions, and the advantages and disadvantages of the different strategies are summarized and discussed. Finally, we analyze the statistics of all the cited works from different points of view and reveal future trends for GAN-based biomedical image registration studies.

## Introduction

The goal of biomedical image registration (BIR) is to estimate a linear or non-linear spatial transformation by geometrically aligning the corresponding anatomical structures between images. The images can be acquired across time, modalities, subjects, or species. By aligning the corresponding structures or mapping the images onto a canonical coordinate space, the registration allows quantitative comparison across the subjects imaged under different conditions. It enables the analysis of their distinct aspects in pathology or neurobiology in a coordinated manner (Oliveira and Tavares, 2014). In addition, image registration is also fundamental to image-guided intervention and radiotherapy.

In recent years, there has been a steady emergence of high-resolution and high-throughput biomedical imaging techniques (Li and Gong, 2012; Chen et al., 2021). Some commonly used macroscale imaging techniques include magnetic resonance imaging (MRI), computed tomography (CT), positron emission tomography (PET), and single photon emission computed tomography (SPECT) (Gering et al., 2001; Staring et al., 2009). However, mesoscale and microscale imaging techniques, such as serial two-photon tomography (STPT) (Ragan et al., 2012), fluorescence micro-optical sectioning tomography (FMOST) (Gong et al., 2016), volumetric imaging with synchronous on-the-fly scan and readout (VISOR) (Xu et al., 2021), and the electron microscope (EM) (Ruska, 1987), play pivotal roles in various neuroscience studies. The resulting exploration of the number, resolution, dimensionality, and modalities of biomedical images not only provides researchers with unprecedented opportunities to study tissue functions, diagnose diseases, etc. but also poses enormous challenges to image registration techniques.

A large number of image registration methods, ranging from the traditional iterative method to the one-shot end-to-end method (Klein S. et al., 2009; Qu et al., 2021), from the fully supervised strategy to the unsupervised strategy (Balakrishnan et al., 2019; He et al., 2021), have been developed to take full advantage of the rapidly accumulating biomedical images with different geometric and modalities. According to the different acquisition techniques of the images, these methods can also be classified into two main categories: mono-modal (intra-modal) registration and cross-modal (or inter-model) registration. Generally, the images of different modalities often vary substantially in their voxel intensity, image texture, and anatomical structures (e.g., due to uneven brain shrinkage resulting from different sample preparation methods). Therefore, cross-modal registration is even more challenging to achieve.

To the best of our knowledge, few studies focus on cross-modal medical image registration. Among the available reviews (Jiang S. et al., 2021), traditional feature-based cross-modal alignment methods have been reviewed in detail. Most of these traditional registration methods are based on iterative training, which is time consuming. The supervised alignment methods are limited by insufficient labels among the learning-based methods. However, unsupervised methods are proposed with various loss functions due to the absence of ground truth and supervision.

Additionally, these unsupervised methods do not perform as well as unimodal on cross-modal images due to too much variation between cross-modal appearances. Nevertheless, efforts are being directed toward removing the modal differences between cross-modalities. Among these various deep-learning-based methods, the Generative Adversarial Networks (GAN) (Goodfellow et al., 2014) have received increasing attention from researchers for their unique network structures and training strategies. In addition, the GAN-based methods have shown extraordinary potential in dealing with cross-modal registration. In particular, the conditional GAN (CGAN) can realize transformation between different styles of images, which provides a new solution to the difficult cross-modal registration method, which has been plagued by the different modality characteristics for a long time.

Since it was proposed, the GAN has been widely used for biomedical image analysis, such as classification, detection, and registration. Its outstanding performance in image synthesis, style translation (Kim et al., 2017; Jing et al., 2020), and the adversarial training strategy has attracted attention in many areas (Li et al., 2021). GAN has been applied to image registration tasks since 2018 (Yan et al., 2018). However, compared with other deep-learning-based registration methods, the GAN-based methods are still in their infancy, and their potential needs further exploration. To the best of our knowledge, there has not yet been a specific review on GAN in biomedical image registration. Therefore, we hereby try to provide an up-to-date and comprehensive review of existing GAN applications in biomedical image registration.

In the survey, we focus on both the GAN-based mono- and cross-modal biomedical image registrations but may highlight more on the contribution of the GAN-based cross-modal image registration. Cross-modal biomedical image registration is still facing many challenges compared with mature mono-modal biomedical image registration.

This review is structured as follows: Section Common GAN structures briefly introduces the basic theory of common GAN related to image registrations; Section Strategies of GAN based biomedical image registration provides a comprehensive analysis of four GAN-based registration strategies; in Section Statistics, we analyze the ratio distribution of some important characteristics of these studies, and in Section Future perspectives, we discuss some open issues and future research perspectives.

## Common GAN structures

This section gives a brief introduction to the GAN structures used for image generation. The structures considered are often used directly or indirectly in the cross-modal biomedical image registration model. We summarize this literature, which considers GAN structures, in Tables 2–5. The section emphasizes the overall architecture, data flow, and objective function of GAN. The differences between the various methods are also presented.

### Original GAN

**Figure 1:**
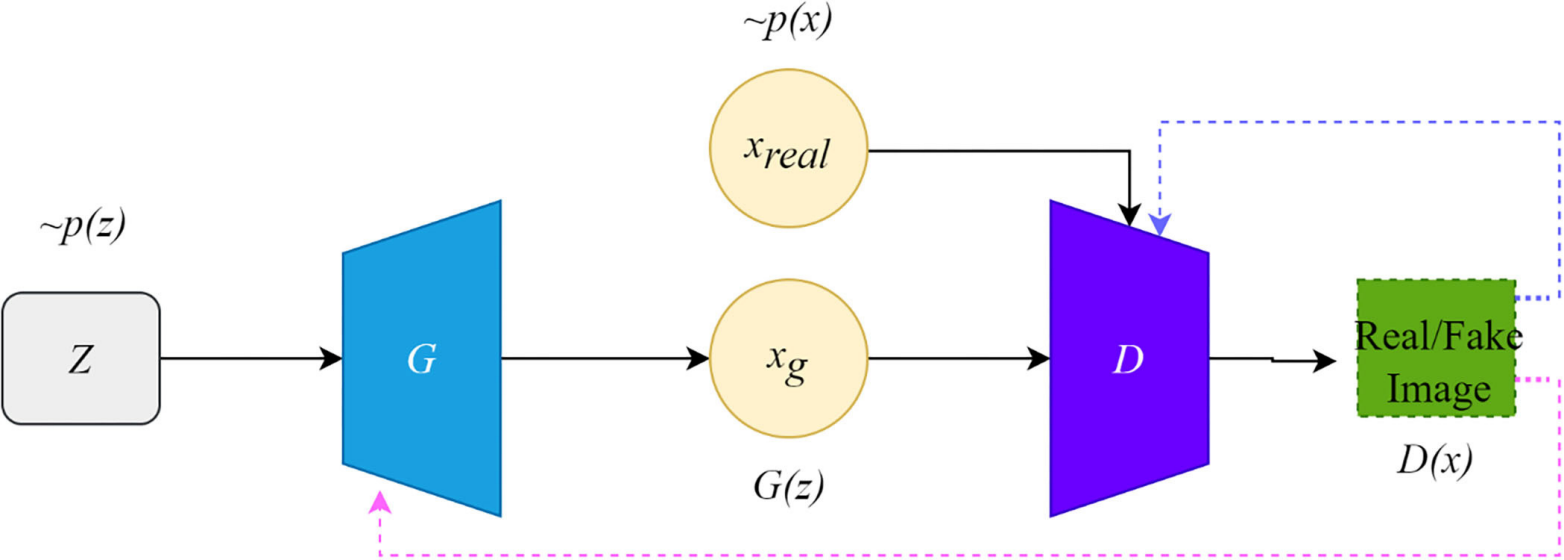
The architecture of the original GAN.

The framework of the original GAN is shown in Figure 1. The original GAN consists of two networks, the generator (G) and the discriminator (D), both of which are fully connected. The input to the generator is a random noise vector from the noise distribution ~*p*(z) (random noise is Gaussian noise or uniform noise). The generator can learn a mapping from the low-dimensional noise vector space to the high-dimensional data space. The input of the discriminator is the real data ~*P*_*r*_*(x)* and the synthetic data ~*P*_*g*_*(x)* by the generator. If the input to the discriminator is real data x, the purpose of the discriminator is to represent the probability that *x* comes from ~*P*_*r*_*(x)* rather than ~*P*_*g*_*(x)*, and the discriminator should classify it as real data and return a value close to 1.

Conversely, if the input is synthetic data, the discriminator should classify it as false data and return a value close to 0. The false signal output from the discriminator is back propagated to the generator to update the network parameters. This framework is trained in an adversarial strategy corresponding to a two-player minimax game. The minimax GAN loss is equivalent to the game's rules, while the generator and the discriminator are equivalent to the two players. The goal of the generator is to minimize the loss by generating synthetic images that look as similar to the real images as possible to fool the discriminator.

In contrast, the discriminator maximizes the loss to maximize the probability of assigning the correct label to both the training examples and the samples from the generator. The training improves the performance of both the generator and the discriminator networks. Their loss functions can be formulated as follows:


$$\begin{array}{lll} L_{D} & = & \underset{D}{max}{𝔼}_{\text{x}\sim {\text{p}}_{\text{d}at\text{a}\left(\text{x}\right)}}\left[logD\left(X\right)\right]+{𝔼}_{\text{z}\sim {\text{p}}_{\text{z}}\left(\text{z}\right)}\left[\log\left(1-D\left(G\left(Z\right)\right)\right)\right] \end{array}$$

(1) $$\begin{array}{lll} L_{G} & = & min_{G}{𝔼}_{\text{z}\sim {\text{p}}_{\text{z}}\left(\text{z}\right)}\left[\log\left(1-D\left(G\left(Z\right)\right)\right)\right] \end{array}$$

where *L*_*D*_ and *L*_*G*_ are the loss functions of *D* and *G*, respectively, and *D* is the binary classifier; it is expected that the data distribution generated by G(z) is close to the real data when the model is optimized.

### DCGAN

**Figure 2:**
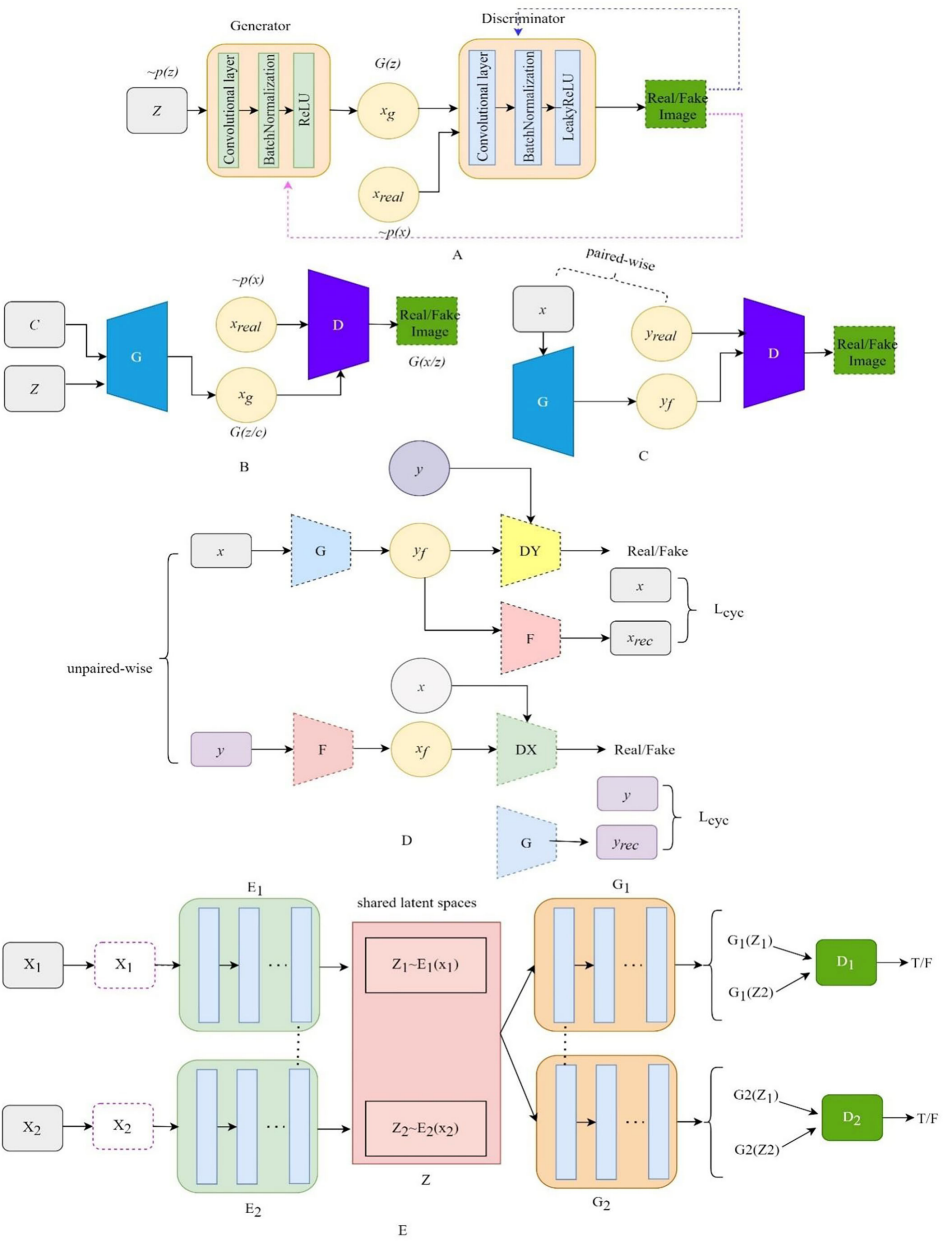
The architecture of the variant GANs. **(A)** The architecture of DCGAN, **(B)** the architecture of CGAN, **(C)** the architecture of Pix2Pix, **(D)** the architecture of CyclGAN, and **(E)** the architecture of UNIT.

Compared with the original GAN, the deep convolutional generative adversarial networks (DCGAN) (Radford et al., 2015) add specific architectural constraints to GAN by replacing all the full-connected neural networks with CNN, which results in stable training. Figure 2A illustrates the structure of DCGAN, in which there are three important changes in the convolutional neural network (CNN) architecture. Firstly, the pooling layers in the discriminator and the generator are replaced by the stridden convolution and the fractionally strung convolutions, respectively, which allow the generator to learn the specific spatial upsampling from the input noise distribution to the output image. Secondly, batch normalization (Ioffe and Szegedy, 2015) is utilized to regulate poor initialization to prevent the generator from collapsing from all samples to a single point. Thirdly, the LeakyReLU (Maas et al., 2013) activation is adopted to replace the maxout activation in all layers of the discriminator, promoting the output of higher-resolution images.

### CGAN

The structure of CGAN (Mirza and Osindero, 2014) is illustrated in Figure 2B. The CGAN performs the conditioning for the output mode by feeding the auxiliary information related to the desired properties *y* and noise vector *z* to the generator and the discriminator. The objective function of the CGAN can be formulated as follows:


$$\begin{array}{lll} L_{D} & = & \underset{D}{max}{𝔼}_{x\sim p_{data}\left(x\right)}\left[logD\left(x|y\right)\right]+{𝔼}_{z\sim p_{z}\left(z\right)}\left[log\left(1-D\left(G\left(z|y\right)\right)\right)\right] \end{array}$$

(2) $$\begin{array}{lll} L_{G} & = & min_{G}{𝔼}_{\text{z}\sim {\text{p}}_{\text{z}}\left(\text{z}\right)}\;\left[\log\left(1-D\left(G\left(z|y\right)\right)\right)\right] \end{array}$$

where *y* is the auxiliary information, which could be a class label, an image, or even the data from different modes. For instance, Pix2Pix (Isola et al., 2017) translates the label image or edge image to an image with another style. InfoGAN (Chen et al., 2016) is regarded as a special CGAN whose condition is a conditional constraint on the random noise *z* for guiding the thickness, slope, and other features of the generated image.

### Pix2Pix

Pix2Pix (Isola et al., 2017) is the first GAN framework for image-to-image translation, which can learn a mapping that transforms an image from one modality to another based on paired-wise images. The structure of Pix2Pix is depicted in Figure 2C. A paired-wise image means that the internal structures in the image pair are accurately aligned, while their texture, brightness, and other modality-related features differ. The objective loss combines CGAN with the *L1* loss so that the generator is also asked to generate images as close as possible to the ground truth:


(3) $$\begin{array}{l} L_{cGAN}\left(G,D\right)=E_{x,y}\left[logD\left(x,y\right)\right]+E_{x,z}\left[\log\left(1-D\left(x,G\left(x,z\right)\right)\right)\right] \end{array}$$

(4) $$\begin{array}{l} L_{L1}\left(G\right)=E_{x,y,z}\left[||y-G\left(x,z\right)|{|}_{1}\right] \end{array}$$

(5) $$\begin{array}{l} G^{*}=arg\begin{array}{c} \min\\ G \end{array}\begin{array}{c} \max\\ D \end{array}L_{cGAN}\left(G,\;D\right)+\lambda L_{L1}\left(G\right) \end{array}$$

where *x* and *y* represent the images from the source and the target domain, respectively; the *L1* loss is a pixel-level metric between the target domain image and the generated image to impose a constraint on *G*, which could recover the low-frequency part of the image; the adversarial loss could recovery the high-frequency part of the image, and λ is the adjustable parameter.

### Cycle-GAN

CycleGAN (Zhu et al., 2017) contains two generators and two discriminators, which are self-bounded by an inverse loop to transform the image between the two domains. The structure of Cycle-GAN is illustrated in Figure 2D. One generator, *G*, translates the source domain image *X* to the target domain image *Y*. Another generator, *F*, learns the inverse mapping of *G*, which brings *G(X)* back to its original image *X*., i.e., *x*→*G(x)*→*F[G(x)]* ≈ *x*. Similarly, for *y* from the domain *Y, F* and *G* also satisfy the cycle-consistent, i.e., *y*→*F(y)*→*G[F(y)]* ≈ *y*. The cycle-consistent loss *L*_*cycle*_ measures the reconstructed image and the real image by pixel-level loss calculation to constrain the training of *G* and *F*, ensuring the consistency of its morphological structure in the transformation process. Two discriminators distinguish between the reconstructed image and the real image. The adversarial loss and the cycle-consistent loss are as follows:


$$\begin{array}{lll} L_{GAN}\left(G,D_{Y}\right) & = & E{\;}_{y\sim p_{Y}\left(y\right)}\left[logD_{Y}\left(y\right)\right] \end{array}$$

(6) $$\begin{array}{l} +E{\;}_{x\sim p_{X}\left(x\right)}\left[logD_{X}\left(x\right)\right] \end{array}$$

$$\begin{array}{l} L_{cyc}\left(G,F\right)\;=𝔼x{\;}_{x\sim p_{X}\left(x\right)}\left[||F\left(G\left(x\right)-x{\left|\right|}_{1}\right)\right] \end{array}$$

(7) $$\begin{array}{l} +𝔼{\;}_{y\sim p_{Y}\left(y\right)}[||G\left(F\left(y\right)-y{\left|\right|}_{1}\right) \end{array}$$

(8) $$\begin{array}{l} L\left(G,F,D_{X},D_{Y}\right)=\;L_{GAN}\left(G,D_{Y}\right)+L_{GAN}\left(F,D_{X}\right)+\lambda \;L_{cyc}\left(G,F\right) \end{array}$$

The training procedure uses the least squares and replays buffer for training stability. UNET (Ronneberger et al., 2015) and PatchGAN (Li and Wand, 2016; Isola et al., 2017) are used to build the generator and the discriminator.

### UNIT

UNIT (Liu et al., 2017) can also perform unpaired image-to-image transformation by combining two variational autoencoder generative adversarial networks (VAEGAN) (Xian et al., 2019), with each responsible for one modality but sharing the same latent space. The UNIT structure is illustrated in Figure 2E, which consists of six subnetworks: two domain image encoders, E1 and E2, two domain image generators, G1 and G2, and two adversarial domain discriminators, D1 and D2. The encoder–generator pair {E1, G1} constitutes a VAE for the X1 domain, called VAE1. For an input image x1∈X1, the VAE1 first maps x1 to a code in a latent space Z via the encoder E1 and then decodes a random-perturbed version of the code to reconstruct the input image via the generator G1. The image x_2_ in X_2_ can be translated to an image in X_1_ by applying G_1_(Z_2_). For real images sampled from the X_1_ domain, D1 should output true, whereas, for images generated by G_1_(Z_2_), it should output false. The cycle-consistency constraint exists in x_1_ = F_2 − 1_[F_1 − 2_(x1)], where F_1 − 2_ = G_2_[E_1_(X_1_)].

## Strategies for GAN-based biomedical image registration

Cross-modal biomedical image registration using GAN has given rise to an increasing number of registration algorithms to solve the current problems mentioned in the introduction section. Based on the different strategies, the algorithms are divided into four categories: modality translation, symmetric learning, adversarial strategies, and joint training. A category overview of the biomedical image registration methods using GAN is shown in Table 1. In the table, we describe the key ideas of the four categories, respectively, and summarize the different implementation methods for each strategy. In the subsequent subsections, we review all the relevant works as classified in Table 1.

**Table 1:** A category overview of biomedical image registration methods using GAN.

**Category**	**Key idea**	**Method**	**Publication**
Modality independent	Translate two different modal-image to the	Modality translation	Tanner et al., 2018; Wei et al., 2019
	same domain, then perform mono-modal		Wei et al., 2020; Xu et al., 2020
	registration		Zhang et al., 2020; Zhou et al., 2021
			Lu et al., 2021
		Latent representation	Mahapatra and Ge, 2020; Yang et al., 2020
		Image decomposition	Qin et al., 2019; Wu and Zhou, 2021
Symmetric Learning	The accuracy of bidirectional registration is optimized by making transformation inverse consistency	GAN	Zheng et al., 2021
		Cyclic-consistency	Lu et al., 2019, 2020
Adversarial learning	Adopt the way of adversarial training to perform image registration. The generator is regarded as registration, and similarity loss is instead of the discriminator	Semi-supervised	Hu et al., 2018; Elmahdy et al., 2019; Li and Ogino, 2019; Luo et al., 2021
		Knowledge distillation	Tran et al., 2022
		Attention mechanisms	Li M. et al., 2021
		Adversarial training	Fan et al., 2018, 2019; Yan et al., 2018
Joint learning	Segmentation, synthesis, and registration network jointly train to improve the performance of each other	Multitask	Mahapatra et al., 2018; Liu et al., 2020; Zhou et al., 2021

To present a comprehensive overview of all the relevant works on GANs in biomedical image registration, we searched science datasets, including Google Scholar, SpringerLink, and PubMed, for all relevant published articles from 2018 to 2021. The keywords included medical image registration/matching alignment, GAN (Generative Adversarial Networks), multimodal (cross-modality) medical image registration, GAN, adversarial medical image registration, segmentation, and registration. About 300 papers are indexed, including 36 papers that completely match our criteria. There are two requirements for our inclusion in the articles. The first is that the topic of the papers is image registration, and the second is that the method is of GAN based on and used to implement the registration strategy. To verify the comprehensiveness of the search, we also searched them separately in the International Conference on Medical Image Computing and Computer-Assisted Intervention (MICCAI), the IEEE International Symposium on Biomedical Imaging (ISBI), and SPIE Medical Imaging to compare with the already searched papers. During the literature review process, we try to integrate all relevant papers to reach a reasonable conclusion; however, because this topic is still in its infancy, the number of published papers is minimal. Therefore, we are unable to conduct an experimental review on this topic because most of the searched articles have no open-source code, and the data are private.

### Modality-Independent based strategy

Biomedical image registration algorithms of modality-independent based strategies mainly focus on cross-modal images. The key idea of the strategy is to eliminate the variance between modalities so that cross-modality registration can be performed on modality-independent data. A modality-independent strategy can be implemented by translating cross-modality, image disentangling, and latent representation methods. This strategy can avoid the design of cross-modal similarity loss. It only uses robust mono-modal similarity loss to guide the optimization of the model. In Table 2, we provide an overview of the important elements of all the reviewed papers. Among these papers, 12 directly use Cycle-GAN as the baseline model, and seven are applied to the registration tasks of MRI-CT, with the organs covered by the brain, liver, retina, and heart.

**Table 2:** Overview of the modality-independent based strategy.

**Publications**	**Organ**	**Method**	**Modality**	**Evaluation metrics**	**Loss**	**Dataset**
Han et al. (2021)	Brain	Cycle-GAN	MRI-CT	M1, 2	L3	–
Fu et al. (2020)	Head and neck	Cycle-GAN	MRI-CT	M2		–
Lu et al. (2020)	Heart	Cycle-GAN	CT-TEE	M1, 3, 4	L1	–
Wei et al. (2020)	Liver	Cycle-GAN	MRI-CT	M1, 2	L3	–
Wei et al. (2019)	Liver	Cycle-GAN	MRI-CT	M1, 2	L3	–
Arar et al. (2020)	/	Cycle-GAN	/	M5	L1, 2	–
Zhang et al. (2020)	Brain	Pix2Pix	T1–T2		L1, 2, 5	D3
Tanner et al. (2018)	Retina and heart	Cycle-GAN	MRI-CT	M1	L1, 3	–
Zhou et al. (2021)	Liver	Cycle-GAN	MRI-CBCT	M1, 10	L3, 4, 5, 6, 8	D8,9
Yang et al. (2020)	Brain	VAE+GAN	MRI-CT	M7, 9	L2, 3	–
Lu et al. (2021)	/	Cycle-GAN, Pix2Pix, Drit, StarGAN-v2	/	M5	/	–
Xu et al. (2020)	Kidney	Cycle-GAN	CT-MR			
Qin et al. (2019)	Lung and brain	CycleGAN	T1–T2	M1, 3, 13	L1, 2, 5, 10	D24
Wu and Zhou (2021)	Brain	CyclegGAN	T1–T2	M14	L1, 3, 9	D25
Mahapatra and Ge (2020)	Lung, brain, retinal	GAN	X-rays- X-rays/T1-T2	M1, 3	L1, 5, 10	D23
Lin et al. (2021)	Brain	RevGAN	MRI-PET	ACC	L1	–

Some brief descriptions of the loss, dataset, and evaluation metrics can be found in Tables 6–8. The symbol–in the last column means that the data used in the paper is a private dataset.

#### Modality translation

**Figure 3:**
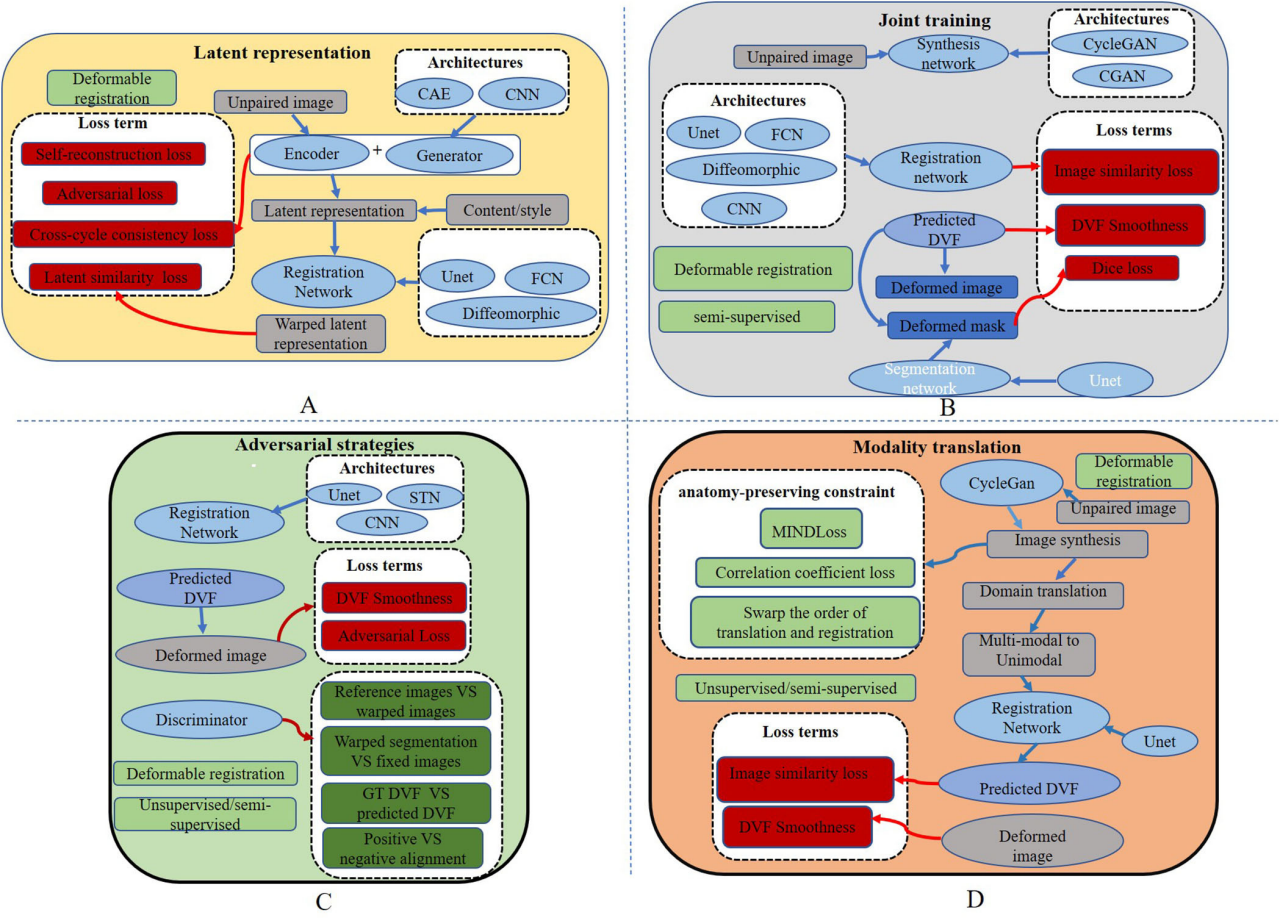
Overall structures of existing cross-modal image registration methods. **(A)** The overall structure of the latent representation method. **(B)** The overall structure of joint learning-based strategy. **(C)** The overall structure of adversarial learning-based strategy. **(D)** The basic structures of modality translation.

To register MRI to CT, Tanner et al. (2018) make the first attempt at modality translation using Cycle-GAN and subsequently perform the mono-modality registration on two images in the same domain. The Patch-GAN uses N × N patches instead of a single value as the output of the discriminator for spatial corresponded-preservation. This pioneering work assessed the feasibility of this strategy. However, the mono-registration significantly relies on the quality of synthetic images. In a subsequent study, to constrain the geometric changes during modality translation, Wei et al. (2020) designed the mutual information (MI) loss to regularize the anatomy and between the classic mono-modal registration method ANTS (Avants et al., 2008, 2009) as the registration network. Xu et al. (2020) combined the deformation field from uni- and multimodal stream networks by dual stream fusion network for cross-modality registration. The uni-modal stream model preserves the anatomy during modality translation using the Cycle-GAN by combining several losses, including the modality independent neighborhood descriptor (MIND) (Heinrich et al., 2012), the correlation coefficient loss (CC), and the L2 loss. The basic structures of these methods are shown in Figure 3D. To further solve the uni-modal mismatch problem caused by the unrealistic soft-tissue details generated by the modality translation, the multimodal stream network is proposed on the UNET-based cross-modal network to learn the original information from both the fixed and moving images. The dual stream fusion network is responsible for fusing the deformation fields of the uni-modal and multimodal streams. The two registration streams' complementary functions preserve the edge details of images. However, the multimodal stream also learns some redundant features, which is not beneficial to the alignment. Unlike the aforementioned methods, Zhou et al. (2021) translate the CBCT and MRI to the CT modality by Cycle-GAN. The UNET-based segmentation network is trained to get the segmentation map of the synthetic CT image for guiding the robust point matching (RPM) registration. The systems combine the synthesis and segmentation networks to implement cross-modality image segmentation. Arar et al. (2020) assume that a spatial translation network (STN) (Jaderberg et al., 2015) registration network (R) and the CGAN-based translation net (T) are commutative, i.e., T°R=R°T. Based on this assumption, the optimized L1-reconstruction loss *L*_*recon*_(*T, R*) = ||*O*_*RT*_−*I*_*target*_||_1_+||*O*_*TR*_−*I*_*target*_||_1_, which encourages T to be geometrically preserved. Benefited from the anatomy-consistency constraints, the registration accuracy can be improved. However, the training of GAN may suffer from non-convergence, which may pose certain additional difficulties to the training of the registration network. Lu et al. (2021) assessed what role image translation plays in the cross-modal registration based on the performance of Cycle-GAN, which also shows the instability of this approach and the overdependence on the data.

#### Image disentangling

Qin et al. (2019) try to learn a registration function in modal-independent latent shape space in an unsupervised manner. The proposed framework consists of three parts: a disentangling image network *via* unpaired modality translation, a deformable registration network in the disentangled latent space, and a GAN modal to learn a similarity metric in the image space implicitly. Several losses are used in the network to train the three parts, including the self-reconstruction loss, the latent reconstruction loss, the cross-cycle consistency, and the adversarial loss with similarity metrics defined in latent space. The work is capable of translating cross-modal images by image disentangling to obtain shape latent representation related to the image alignment. This method can alleviate unrealistic image generation from the Cycle-GAN-based approaches. However, the deformation field generated by latent shape representation introduces unsmooth edges. Wu and Zhou (2021) propose a fully unsupervised registration network through image disentangling. The proposed registration framework consists of two parts: one registration network aligns the image from x to y, and the other aligns the image from y to x. Each part consists of two subnetworks: an unsupervised deformable registration network and a disentangling representation network *via* unpaired image-to-image translation. Unlike Qin et al. (2019), the representation disentangling model aims to drive a deformable registration network for learning the mapping between the two modalities.

#### Latent representation

Mahapatra and Ge (2020) use a convolutional autoencoder (CAE) network to learn latent space representation for different modalities of images. The generator is fed into latent features from CAE to generate the deformation fields. The intensity and shape constraints are achieved by content loss, including the normal mutual information (NMI), the structural similarity index measure (SSIM) (Wang et al., 1987, 2004), and the visual graphics generator (VGG) (Simonyan and Zisserman, 2014) with L2 loss. The cycle consistency loss and the adversarial loss are used to constrain the deformation field consistency, which is calculated as follows:


(9) $$\begin{array}{l} L\left(G,F,D_{I^{Flt}},D_{I^{Ref}}\right)=L_{adv}+L_{content}+\lambda L_{cyc}\left(G,F\right) \end{array}$$

$$\begin{array}{l} L_{content}\left(I^{Trans},I^{Ref}\right)\\ \;=NMI\left(I^{Ref},I^{Trans}\right)+\left[1-SSIM\left(I^{Ref},I^{Trans}\right)\right]\\ \;+VGG\left(I^{Ref},I^{Trans}\right) \end{array}$$

(10) $$\begin{array}{l} L_{cyc}\left(G,F\right)=E_{x}||F\left(G\left(x\right)\right)-x|{|}_{1}+E_{y}||G\left(F\left(x\right)\right)-y|{|}_{1} \end{array}$$

$$\begin{array}{lll} L_{adv} & = & \;L_{cycleGAN}\left(G,D_{I^{Ref}}\right)+L_{cycleGAN}\left(F,D_{I^{Flt}}\right) \end{array}$$

(11) $$\begin{array}{l} +log\left(1-MSE_{Norm}\left(I^{Def-APP},I^{Def-Recv}\right)\right) \end{array}$$

where (*G, F)* represents the two generators, $D_{I^{Flt}}$ and $D_{I^{Ref}}$ represent *I*^*Flt*^ and *I*^*Ref*^ as the real data of the discriminator, *x* and *y* represent the original images of the two modalities, and *MSE*_*Norm*_ is the MSE normalized to [0, 1]. Yang et al. (2020) transform image modality through a conditional auto-encoder generative adversarial network (CAE-GAN), which redesigns VAE (Kingma and Welling, 2013) and GAN to form the symmetric UNET. The registration network uses a traditional nonparametric deformable method based on local phase differences at multiple scales. The overall structure of the latent representation method is shown in Figure 3A.

### Symmetric learning-based strategy

Table 3 lists two papers about using the symmetric learning-based GAN methods. Both of them perform image registration for CT-MRI. One is used on the brain, and another on the heart.

**Table 3:** Overview of symmetric learning-based methods.

**Publication**	**Organ**	**Method**	**Modality**	**Evaluation metrics**	**Loss**	**Dataset**
Zheng et al. (2021)	brain	GAN	CT-MRI	M1	L1, 2	D3, 4, 5, 6, 7
Lu et al. (2019)	heart	Cycle-GAN	MRI-CT	M1	L3, 7	D2

Some brief descriptions of the loss, dataset, and evaluation metrics can be found in Tables 6–8.

#### Cyclic-consistency

From the perspective of cyclic learning, symmetric learning can assist and supervise each other. The CIRNet (Lu et al., 2019) uses two cascaded networks with identical structures as the symmetric registration networks. The two cascaded networks share the weights. The L2 loss is used as L*cyc* to enforce image A translating through two deformation fields ϕ1 and ϕ2, and A(ϕ1, ϕ2) = A. L*cyc* is defined as follows:


(12) $$\begin{array}{l} L_{cyc}\left(A\left(\varphi_{1},\varphi_{2}\right),A\right)=\;\frac{1}{N}\underset{i\in \Omega }{\sum }{\left(A\left(\varphi_{1},\varphi_{2}\right)\left(i\right),A\left(i\right)\right)}^{2} \end{array}$$

where *N* represents the number of all the voxels, and Ω refers to all the voxels in the image. The total loss of the two registration networks is represented by Equations (13, 14), respectively:


$$\begin{array}{lll} L_{R1}\left(A,B\right) & = & -L_{NCC}\left(A\left(\varphi_{1}\right),B\right)+\alpha L_{reg}\left(\varphi_{1}\right) \end{array}$$

(13) $$\begin{array}{l} +\beta L_{cyc}\left(A\left(\varphi_{1},\varphi_{2}\right),A\right) \end{array}$$

$$\begin{array}{lll} 2\left(A\left(\varphi_{1}\right),A\right) & = & -L_{NCC}\left(A\left(\varphi_{1},\varphi_{2}\right),A\right)+\alpha L_{reg}\left(\varphi_{2}\right) \end{array}$$

(14) $$\begin{array}{l} +\beta L_{cyc}\left(A\left(\varphi_{1},\varphi_{2}\right),A\right) \end{array}$$

(15) $$\begin{array}{l} L_{reg}\left(\varphi_{1}\right)=\underset{i\in \Omega }{\sum }{\nabla \varphi \left(i\right)}^{2} \end{array}$$

where *A*(φ_1_) denotes a warped image produced by the module R1, *A*(φ_1_, φ_2_) denotes a warped image produced by the module R2, and *L*_*reg*_(φ_1_) is the smooth regularization*L*_*reg*_.

SymReg-GAN (Zheng et al., 2021) proposes a GAN-based symmetric registration to resolve the inverse-consistent translation between cross-modal images. A generator performs the modality translation, consisting of an affine-translation regressor and a non-linear-deformation regressor. The discriminator distinguishes between the translation estimation and the ground truth. The SymReg-GAN is trained by jointly utilizing labeled and unlabeled images. It encourages symmetry in registration by enforcing a condition that in the cycle composed of the transformation from one image to the other, its reverse transformation should bring the original image back. The total loss combines the symmetry loss, registration loss, and supervision loss into one. This method takes full advantage of both labeled and unlabeled data and resolves the limitation of iterative optimization by non-learning techniques. However, the spatial transformation and the modality transformation may not be the same, and even if the spatial transformation is symmetric, the transformation error may still be cyclic.

### Adversarial learning-based strategy

Biomedical image registration algorithms of adversarial learning-based strategies utilize adversarial loss to drive the learning of registration networks such as GAN. Adversarial loss consists of two parts: the training aim of the generator is to generate an image that makes the discriminator consider it real, and the optimization objective of the discriminator is to distinguish between an image generated by the generator and a real image in the dataset as accurately as possible. Based on this strategy, several methods, as shown in Table 1, including semi-supervised, knowledge distillation, attention mechanisms, and adversarial training, are implemented to improve registration performance. The similarity loss is instead by learning a discriminator network. Although GAN can be trained unsupervised, paired training data may be more helpful for model convergence for the cross-modal registration modal. An overview of the crucial elements of all the reviewed papers is shown in Table 4. Five papers are for mono-modal image registration and four for cross-modal registration. The overall structure of this strategy is shown in Figure 3C.

**Table 4:** Publications of adversarial strategy based.

**Publications**	**Organ**	**Method**	**Modality**	**Evaluation metrics**	**Loss**	**Dataset**
Tran et al. (2022)	Liver, brain	GAN	CT-CT/MRI-MRI	M1, 11	L1, 2	D8, 9, 10, 11, 12, 13, 14, 15, 16, 17
Li and Ogino (2019)	Liver	GAN	/	M1, 2	L1, 2, 5	
Hu et al. (2018)	Prostate	GAN	MRI-TRUS			
Bessadok et al. (2021)	Brain	GAN	MRI-MRI	M5, 12	L1	D15
Li M. et al. (2021)	Brain	GAN	MRI-PET	M1	L2, 5	D29
Fan et al. (2018)	Brain	GAN	MRI-MRI	M1, 4	L1	D18
Fan et al. (2019)	Brain	GAN	MRI-MRI	M1, 4	L1	D18
Yan et al. (2018)	Rectum	GAN	MRI-TRUS	M1, 2	L1	
Tran et al. (2022)	Prostate cancer	GAN	MRI-TRUS	M2	L1	
Luo et al. (2021)	Lung	GAN	X-rays-X-rays	M1, 3, 4, 11	L6, 7, 9	D20

A brief description of the loss, dataset, and evaluation metrics can be found in Tables 6–8. The symbol–in the last column means that the data used in the paper is a private dataset.

#### Adversarial training

For image registration tasks, the effect of adversarial loss is to make the warped image closer to the target image (Yan et al., 2018). The role of the generator is to generate a deformation field, and the task of the discriminator is to identify the alignment image. For more stable loss, Wasserstein GAN (WGAN) is adopted. Since a discriminator can be considered a registration image quality assessor, the quality of a warped image can be improved with cross-modal similarity metrics. However, the training of GAN may suffer from non-convergence, which may pose additional difficulties in training the registration network. Compared to Fan et al. (2018, 2019) and Yan et al. (2018) select more reasonable reference data for training the discriminator for better model convergence.

#### Semi-supervised

Hu et al. (2018) use a biomechanical simulation deformation field to regularize the deformation fields formed by the alignment network. The generator is fed into simulated motion data to form a translation. The discriminator tries to distinguish the dense displacement field from ground truth deformation. Another similarity loss metric warps moving labels and fixed labels in a weakly supervised manner. Li and Ogino (2019) propose a general end-to-end registration network in which a CNN similar to UNET is trained to generate the deformation field. For better guiding, the anatomical shape alignment and masks of moving and fixed objects are also fed into the registration network. The input of the discriminator net is a positive and negative alignment pair, consisting of masks and images of the fixed and warped images for guiding a finer anatomy alignment. In addition, an encoder extracts anatomical shape differences as another registration loss. The studies by Elmahdy et al. (2019) and Luo et al. (2021) are similar to that of Li and Ogino (2019), in which the adversarial learning-based registration network joint segmentation and registration with segmentation label information as the input of the generator and the discriminator. The dice similarity coefficient (DSC) and the normalized cross-correlation (NCC) are added to the generator to avoid slow convergence and suboptimal registration.

#### Knowledge distillation

Knowledge distillation is a process of transferring knowledge from a cumbersome pre-trained model (i.e., the teacher network) to a light-weighted one (i.e., the student network). Tran et al. (2022) used knowledge distillation by adversarial learning to streamline the expensive and effective teacher registration model to a light-weighted student registration model. In their proposed method, the teacher network is the recursive cascaded network (RCN) (Zhao et al., 2019a) for extracting meaningful deformations, and the student network is a CNN-based registration network. When training the registration network, the teacher network and the student network are optimized by *L*_*adv*_ = Υ*l*_*rec*_+(1−Υ)*l*_*dis*_, where *l*_*rec*_ represents the reconstructed loss and the discriminator loss, and *l*_*rec*_ and *l*_*dis*_ are expressed as follows:


(16) $$\begin{array}{l} l_{rec}\left(I_{m}^{h},I_{f}\right)=1-CorrCoef\left[I_{m}^{h},I_{f}\right] \end{array}$$

(17) $$\begin{array}{l} l_{dis}=||D_{\theta }\left(\emptyset_{s}\right)-D_{\theta }\left(\emptyset_{t}\right))|{|}_{2}^{2}+\lambda {\left(||\nabla_{\hat{\emptyset_{s}}}D_{\theta }\left(\hat{\emptyset_{s}}\right)|{|}_{2}-1\right)}^{2} \end{array}$$

(18) $$\begin{array}{l} \hat{\emptyset_{s}}=\beta \emptyset_{t}+\left(1-\beta \right)\emptyset_{s} \end{array}$$

where $I_{m}^{h}$ denotes a warped image by the student network, *CorrCoef*[*I*_1_−*I*_2_] is the correlation between images I1 and I2, ∅_*s*_ and ∅_*t*_ denote the deformation of the teacher and the student networks, respectively, and$\;\hat{\emptyset_{s}}$ denotes the joint deformation. Applying knowledge distillation by means of adversarial learning provides a new and efficient way to reduce computational costs and achieve competitive accuracy.

#### Attention mechanisms

To reduce feature loss of the upsampling process in a registration network, Li M. et al. (2021) proposed a GAN-based registration network combining UNET with dual attention mechanisms. The dual attention mechanisms consist of the channel attention mechanism and the location attention mechanism. Meanwhile, the residual structure is also introduced into the upsampling process for improving feature restoration.

### Joint learning-based strategy

Joint learning of segmentation, registration, and synthesis networks can improve their performance for each other. An overview of the essential elements of all the reviewed papers is shown in Table 5. Two of which are for mono-model registration methods. The overall structure is shown in Figure 3B.

**Table 5:** Joint learning-based methods and publications.

**Publication**	**Organ**	**Method**	**Modality**	**Evaluation metrics**	**Loss**	**Dataset**
Liu et al. (2020)	Liver tumor	CGAN	CT-CT	M1, 3, 4	L1,2	–
Zhou et al. (2021)	Liver tumor	CycleGAN	MRI-CBCT	M1, 10	L3, 4, 5, 6, 8	D8
Mahapatra et al. (2018)	Lung	CycleGAN	X-rays- X-rays	M1, 10	L1, 3, 9	D23

A brief description of the loss, dataset, and evaluation metrics can be found in Tables 6–8. The symbol–in the last column means that the data used in the paper is a private dataset.

**Table 6:** A brief summary of different losses used in the reviewed publications in Tables 2–5.

**Abbre**	**Loss**	**Name**	**Remark**
L1	L_adv_	23 adversarial learning loss	The discriminator introduces the adversarial loss to distinguish synthetic data from real data, consisting of cross-entropy loss and least squares loss
L2	L_pix_	Pix-level supervision loss	Pix-level loss evaluates the different intensity values, consisting of L1, L2, and Frobenius norm
L3	L_cyc_	Cycle consistency loss	Element-wise loss measures the self-similarity of the image of cycled translation to the source domain image when training with an unpaired image from two domains
L4	L_mind_	Modality-independent neighborhood descriptor	The pixel-level similarity metric is used to measure the structural similarity between two different modal images
L5	L_cc_	Correlation coefficient loss	Structural similarity metrics between two different modal images
L6	L_seg_	Segmentation loss	Measuring the difference between a segmented prediction label and a ground truth label
L7	L_Ncc_	Normalized cross-correlation	Used for mono alignment tasks to measure the level of alignment of the warped image to the fixed image
L8	L_idt_	Identity loss	Identity loss regularizes the generators to be near an identity mapping when real samples of the target domain are provided
L9	DM	Hamming distance	Pix-level similarity metrics for image feature focus on a hash value
L10	L_lat_	Latent reconstruction loss	Similarity measure of latent spatial features
L11	L_Leastsquares_	Least squares loss	Used in generators and discriminators as adversarial loss

The L in the first column represents the loss.

**Table 7:** A brief summary of different metrics, which are all in respect to the ground truth.

**Abbr**	**Metrics**	**Remarks**
M1	DICE, Median DSC	The dice coefficient calculates the degree of overlap between the aligned image and the ground truth
M2	TRE (Targeted registration error)	TRE represents the distance sum of the corresponding landmark point between the target image and the aligned image
M3	HD (Hausdorff distance)	HD computes the distance between the contour of the predicted segmentation region and the ground truth to measure shape similarity
M4	ASD, ASSD (Average symmetric surface distance)	Average symmetric surface distance metrics for measuring image alignment
M5	AE (Average euclidean)	Pixel-level description of the distance between the aligned image and the fixed image to measure the similarity
M6	RMSE (Root mean square error)	Used in alignment to describe the deviation of pixels between the aligned image and the real image
M7	MI (Mutual information)	A metric commonly used for cross-modal registration
M8	NMI (Normal mutual information)	Used to measure intensity consistency of images
M9	SLPD (Sum of local phase differences)	Measure the similarity by the sum of local phase differences
M10	Mean ± Std	Means and standard deviations between two images
M11	Jcd (Jaccard)	Used in alignment tasks to describe the dissimilarity between images
M12	PCC (Pearson correlation coefficient)	Like M5, but is more suitable in higher dimensions
M13	MCD (Mean contour distance)	Measure the similarity of images by Mean Contour Distance
M15	MNCC (Mean normalized cross correlation)	Mean Normalized correlation between two images
M16	SSIM	Metrics the structural similarity with respect to a given ground truth

The M in the first column indicates metrics.

**Table 8:** Common datasets used in the reviewed literature.

**Abbre**	**Dataset**	**Anatomy**	**Purpose**	**Modality**
D1	Abdomen (ABD)	Kidney	Healthy abdominal organ segmentation	CT, MR
D2 (Bernard et al., 2018)	ACDC	Heart	Heart segmentation	MRI
D3 (Bakas et al., 2018)	(BraTS) 2018	Brain	Brain tumor segmentation	MRI
D4 (Gousias et al., 2012)	ALBERTs	Newborn brain	Manual segmentation of labeled atlases	MRI
D5	LPBA40	Brain	Medical image registration for Continuous Registration Challenge	MRI
D6	IBSR18	Brain	Medical image registration for Continuous Registration Challenge	T1-weighted
D7	CUMC12 and MGH10	Brain	Medical image registration for Continuous Registration Challenge	MRI
D8 (Bilic et al., 2019)	LiTS	Liver	Liver segmentation	CT
D9 (Kavur et al., 2021)	CHAOS	Liver	Liver segmentation	CT
D10 (Antonelli et al., 2022)	MSD	Liver	Liver tumor segmentation	CT
D11 (Zhao et al., 2019b)	BFH	Liver	Liver tumor segmentation	CT
D12 (Heimann et al., 2009)	SLIVER	Liver	Liver segmentation	CT
D13	LSPIG	Liver	Liver Segmentation of Pigs	CT
D14 (Mueller et al., 2005)	ADNI	Brain	Brain MRI	MRI
D15 (Di Martino et al., 2014)	ABIDE	Brain	Toward a large-scale evaluation of the intrinsic brain architecture in autism	R-FMRI
D16 (Bellec et al., 2017)	ADHD	Brain	Brain MRI	MRI
D17 (Shattuck et al., 2008)	LPBA	Brain	Brain MRI	MRI
D18 (Klein A. et al., 2009)	•LPBA40 •IBSR18 •CUMC12 •MGH10	Brain	Medical image registration for Continuous Registration Challenge	MRI
D20 (Shiraishi et al., 2000)	JSRT	Chest radiographs	Radiologists' detection of pulmonary nodules	x-ray
D2 (Candemir et al., 2013)	MONT	Chest radiographs	Lung segmentation	x-ray
D22 (Jaeger et al., 2013)	SHEN	Chest radiographs	Automatic tuberculosis screening	x-ray
D23 (Wang et al., 2017)	NIH ChestXray14	Chest	Classification studies	x-ray
D24 (Menze et al., 2014)	BraTS'2017	Brain	The Brain Tumor Segmentation	MRI
D25	IXI	Brain	Analysis of brain development	MR
D27	•RIRE •Atlas	Brain	image registration evaluation	CT, MR
D28 (LaMontagne et al., 2019)	OASIS-3	Brain	Cognitive Dataset for Normal Aging and Alzheimer's Disease	MR

#### Multitask

Existing experiments show that a segmentation map can help registration by joint learning. However, in real registration tasks, segmentation labels may not be available. Liu et al. (2020) propose a joint system of segmentation, registration, and synthesis *via* multi-task learning. The objectives of the CGAN-based synthesis model and the registration model are optimized *via* a joint loss. The segmentation network is trained in a supervised manner. The segmentation module estimates the segmentation map for the moving, fixed, and synthesized images. During the training procedure, a dice loss is optimized between the segmentation maps of the warped moving image and the fixed image. The result proves that the segmentation task can improve registration accuracy. Zhou et al. (2021) take advantage of each other through the joint Cycle-GAN and UNET-based segmentation network to solve the missing label problem *via* Cycle-GAN's translating of the two modalities to the third one with a large number of available labels. Thus, the synthesis network improves the segmentation accuracy and further improves the accuracy of the RPM registration. Mahapatra et al. (2018) have trained the generation network to complete the alignment of the reference and moving images by combining the segmentation map that is used directly as the input to the generator, with no need to train an additional segmentation network. Segmentation and alignment are mutually driven. The ways of joining image registration task and image segmentation task may improve the accuracy by sharing the result of learning, which can expand the goal of the registration research.

## Statistics

**Figure 4:**
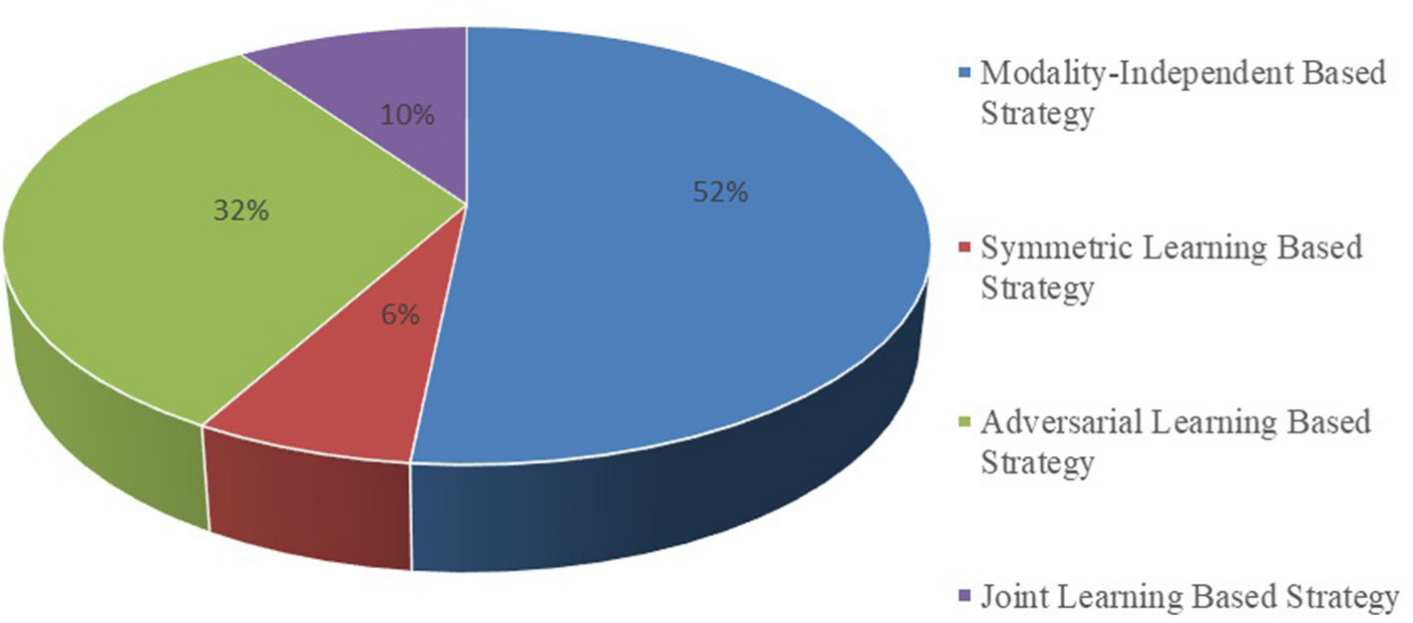
Proportional distribution pie chart of the number of publications on different implementation strategies.

**Figure 5:**
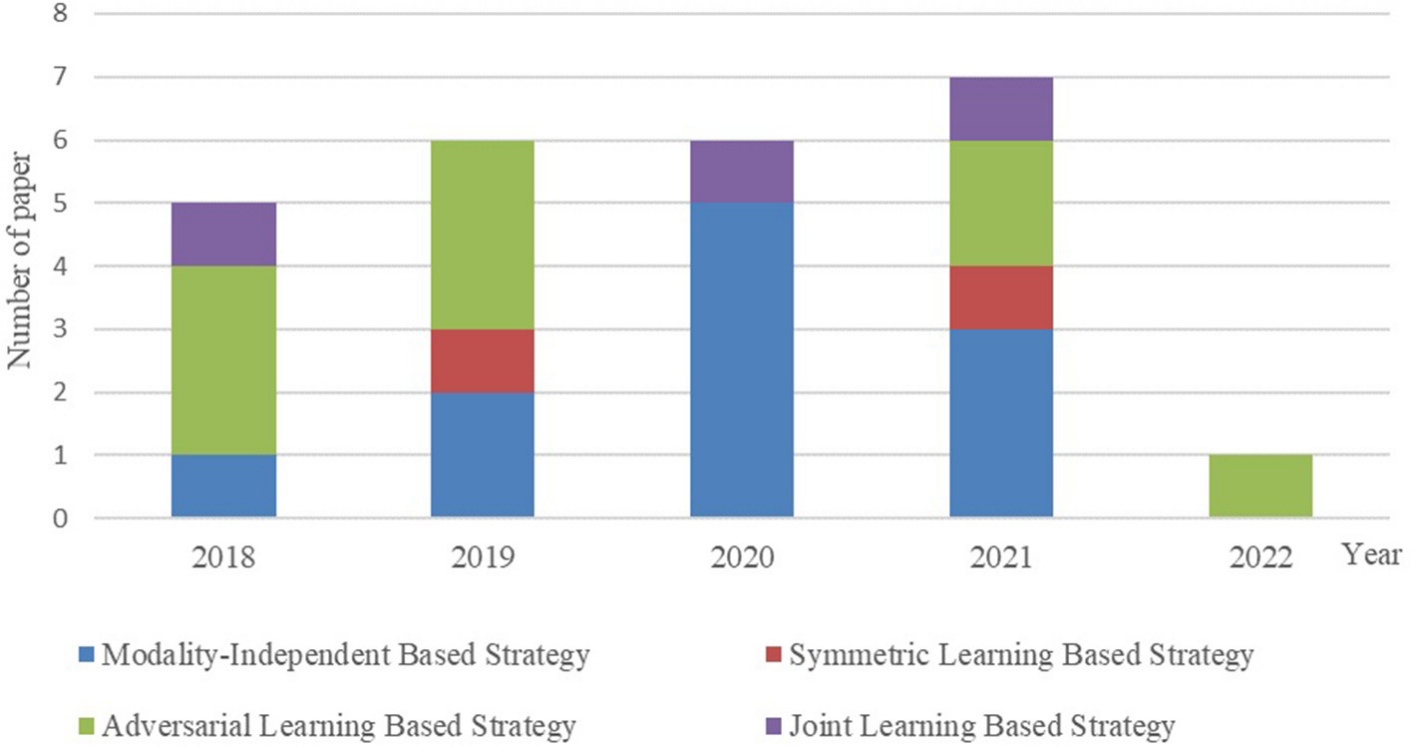
Contrast bar graph of the number of publications on the four strategies for the GAN-based biomedical image registration over the last 5 years from 2018–2022.

**Figure 6:**
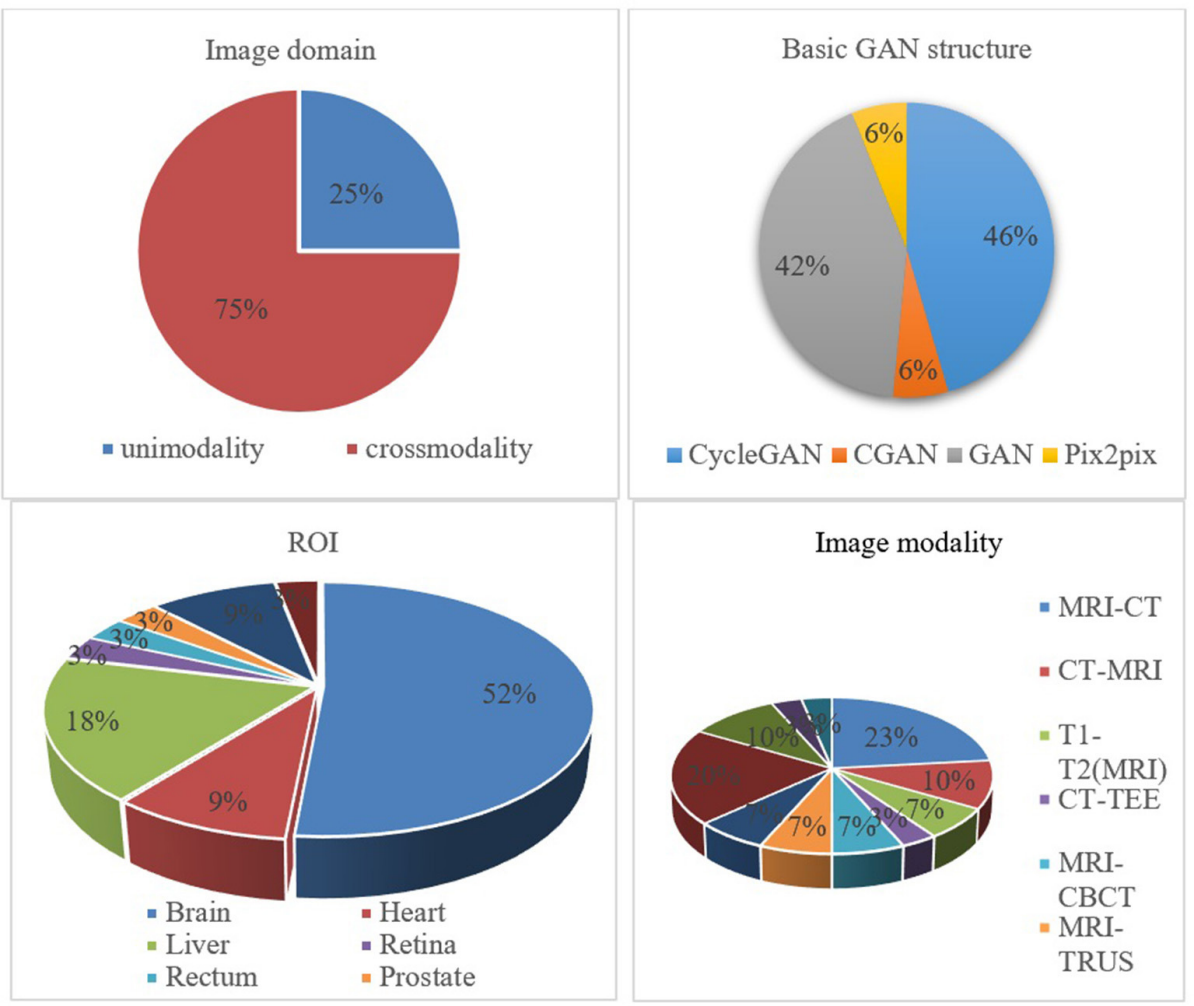
Percentage pie chart of various attributes of the GAN-based biomedical image registration methods.

It is essential to conduct relevant analyses from a global perspective after a detailed study of each category of biomedical image registration strategies. In the past 4 years, more than half of the reviewed works have used the modality-independent-based strategy to solve cross-modal biomedical registration—the methods of Adversarial Learning Based Strategy account for 32%. From 2020 to 2021, the number of articles published on the modality-independent-based strategy was higher than others, peaking in 2020. However, there is a drop-down trend in 2021. As noted, no paper on the adversarial learning-based strategy was published in 2020. In the other years, the works on the adversarial learning-based strategy are published in a balanced proportion, with the detailed percentages shown in Figures 4, 5. In addition to analyzing the trends in the published number of papers and the popularity of the four strategy categories, we also analyzed the percentage distribution of the other characteristics, which is shown in Figure 6. A total of 75% of the works aim to solve the problem of the cross-modal domain of biomedical image registration, among which 46 and 42% adopt direct or indirect Cycle-GAN and GAN are part of the important structure of the registration framework. Cycle-GAN is utilized only for the cross-modal domain of biomedical image registration, whereas GAN is utilized for both cross-modal and uni-modal image registration. In the cross-modal bioimage registration, 33% of the works perform image registration between CT and MRI. The number of articles using MRI accounts for 97%. Regarding the region of interest (ROI), the brain and liver are the most studied sites. The brain is the top registration target in all works. The reason for the wide adoption of the brain consists of its clinical importance, availability in public datasets, and relative simplicity of registration.

**Figure 7:**
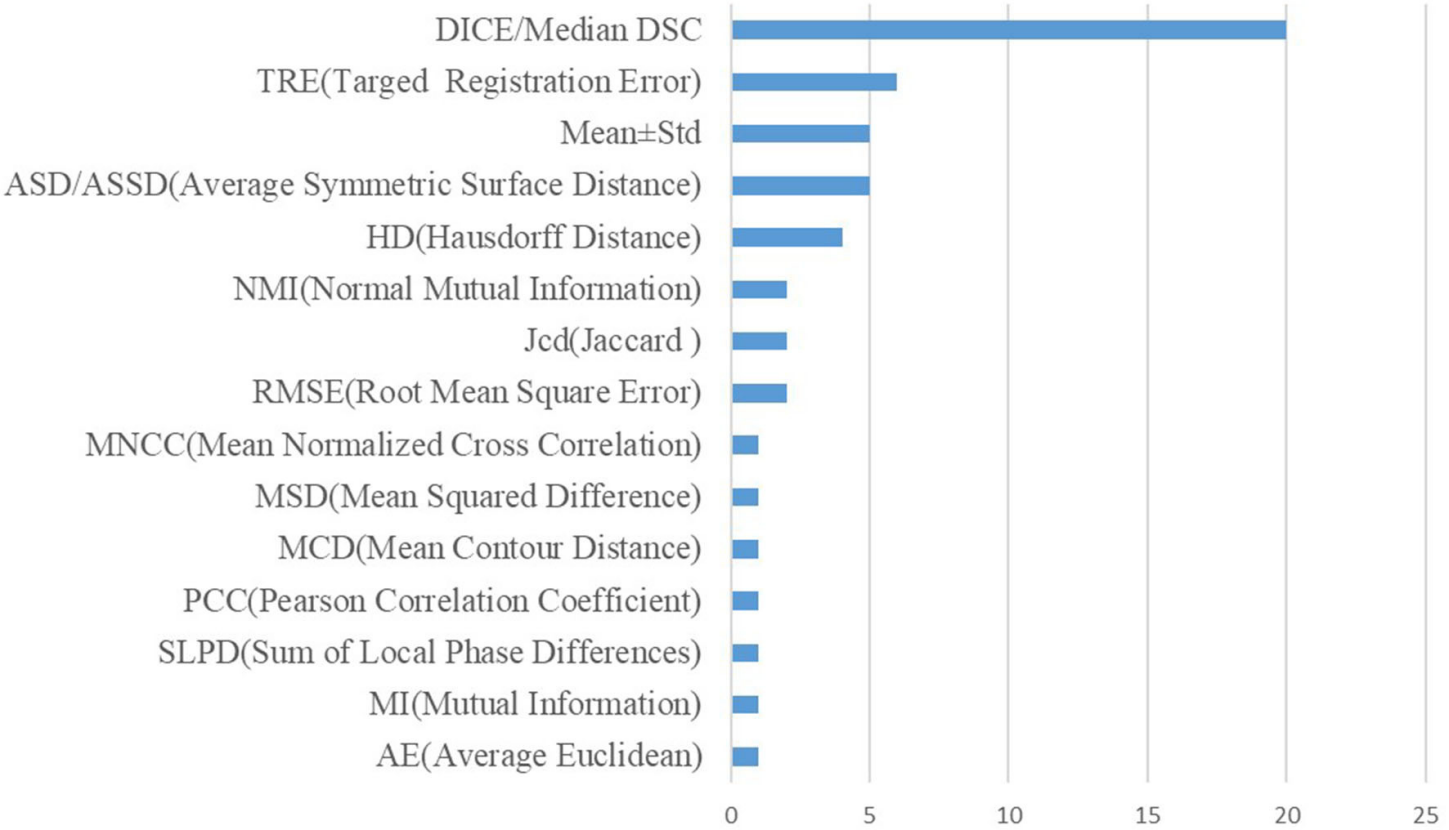
Performance metrics statistics of existing registration methods.

In addition, the metrics used in the cross-modal registration methods are shown in Figure 7. As seen in the figure, dice and TRE are the top two most frequently used metrics. The Dice coefficient calculates the degree of overlap between the aligned image and the ground truth, and the confusion matrix formula is as follows:


(19) $$\begin{array}{l} Dice=\;\frac{2^{*}\left(ref\cap w\right)}{ref\cup w} \end{array}$$

where “ref” refers to the reference image, and “w” represents a warped image. Obviously, when the two images overlap exactly, the Dice coefficient is 1. TRE represents the distance sum of the corresponding landmark between the target image and the aligned image and is expressed as follows:


(20) $$\begin{array}{l} TRE=\;\frac{1}{n}\overset{n}{\underset{i=1}{\sum }}|d_{i}^{r}-d_{i}^{w}| \end{array}$$

where *n* is the number of landmarks, *r* is the reference image, *w* is the aligned image, *i* is the *i*-th corresponding point, and *d* indicates the Euclidean distance.

## Future perspectives

### Exploring in-between representatives of two modalities

Many existing modality-translation-based methods for cross-modality biomedical image registration rely on synthetic images to train the mono-modal registration network because it is difficult to develop cross-modality similarity measures. Although such a training scheme does not need to perform cross-modal similarity metrics to improve the image synthesis performance, it is still necessary to design various losses to constrain other feature changes. Additionally, is the synthetic modality useful for improving registration performance? As far as we know, this intensity information does not play a key role in improving image performance. Some shape features, such as edges and corners, are essential for image registration. An in-between representation was found (Lu et al., 2021), i.e., COMIR, which maps the modalities to their established “common ground.” An in-between representative with characteristics relevant to the accurate alignment would be good. In the future, more workers are expected to be carried out in this direction to find the in-between representatives.

### Exploring quality assessment guided modal translation network

The image quality generated by the mode translation network directly affects the accuracy of the registration algorithm. Therefore, an important research direction is how to effectively and reasonably evaluate the quality of images generated by the GAN network. Additionally, an effective generated image quality evaluation method can be used to constrain the mode translation network's training process and improve the modal translation's effectiveness. There have recently been quality evaluation methods for images generated by GAN (Gu et al., 2020), but there is still a lack of quality evaluation methods for synthetic biomedical images.

### Designing large-scale biomedical image generation GAN network

The size of images existing image generation networks can generate is minimal (Brock et al., 2019), but biomedical images are generally of high resolution, especially biological images used in neurological research. The training process of the existing GAN network is difficult to converge, especially with the increase in image size. The dimension of data space will dramatically increase. This challenge is difficult with current hardware levels and GAN-based image-synthesized methods. Therefore, designing an image synthesis network capable of synthesizing large-scale biomedical images is also a future direction.

### Designing prior knowledge-guided registration methods

Traditional image registration models often use some standard landmarks like points and lines as guidance to optimize the model. Several recent studies have shown that a segmentation mask can be utilized in the discriminator (Luo et al., 2021) or generator (Mahapatra et al., 2018) for guiding the edge alignment. However, their works simply use only a segmentation mask as the edge space correspondence guidance. More space correspondence features are expected to be explored and verified.

## Conclusions

This paper provides a comprehensive survey of cross-modal and mono-modal biomedical image registration approaches based on GAN. The commonly used GAN structures are summarized, followed by the analyses of the biomedical image registration studies of the modality-independent based strategy, the symmetric learning-based strategy, the adversarial learning-based strategy, and the joint learning-based strategy from different implementation methods and perspectives. In addition, we have conducted a statistical analysis of the existing literature in various aspects and have drawn the corresponding conclusions. Finally, we outline four interesting research directions for future studies.

## Author contributions

TH, JW, and LQ contributed to the conception and design of this paper. TH completed the literature selection and the writing of the first draft. JW and ZJ improved the writing of the paper. LQ provided constructive comments and determined the final draft of the paper. All authors contributed to the drafting of the manuscript. All authors contributed to the article and approved the submitted version.

## Funding

This research was funded by the National Natural Science Foundation of China (61871411, 62271003, and 62201008), the Sci-Tech Innovation 2030 Agenda (2022ZD0205200 and 2022ZD0205204), the University Synergy Innovation Program of Anhui Province (GXXT-2021-001), and the Natural Science Foundation of the Education Department of Anhui Province (KJ2021A0017).

## Conflict of interest

The authors declare that the research was conducted in the absence of any commercial or financial relationships that could be construed as a potential conflict of interest.

## Publisher's note

All claims expressed in this article are solely those of the authors and do not necessarily represent those of their affiliated organizations, or those of the publisher, the editors and the reviewers. Any product that may be evaluated in this article, or claim that may be made by its manufacturer, is not guaranteed or endorsed by the publisher.
