# Supplementary material for: Review of Generative Adversarial Networks in mono- and cross-modal biomedical image registration
Source: Front Neuroinform. 2022 Nov 22;16:933230. doi: 10.3389/fninf.2022.933230 (PMC9724825; doi:10.3389/fninf.2022.933230)
Supplement: Supplementary file 1 [file Data_Sheet_1.pdf]

Appendix

Links to download the datasets that appear in the review articles are listed in the following appendix, with the full names of the datasets given in the second column. The number in the parentheses in the first column is the number of articles citing this dataset in the above review.

Dataset	Fullname	Link
**Brain**		
ACDC (1)	Automated Cardiac Diagnosis Challenge	https://acdc.creatis.insalyon.fr/#challenge/584e75606a3c77492fe91bba
BraTS2018 (2)	Brain tumor segmentation	https://www.med.upenn.edu/sbia/brats2018/data.html
ALBERT (1)		www.brain-development.org
LPBA40 (1)	LONI Probabilistic Brain Atlas Individual Subject Data	https://www.loni.usc.edu/research/atlas_downloads
IBSR18 (1)	The Internet Brain Segmentation Repository	https://continuousregistration.grand-challenge.org
CUMC12 (1)	Columbia University Medical Center	https://continuousregistration.grand-challenge.org
MGH10 (2)	Massachusetts General Hospital	https://continuousregistration.grand-challenge.org
ADNI (1)	Alzheimer's Disease Neuroimaging Initiative	https://adni.loni.usc.edu/data-samples/access-data/
ABIDE (2)	Autism brain imaging data exchange	http://fcon_1000.projects.nitrc.org/indi/abide/
BraTS2017 (1)	Brain tumor segmentation	https://paperswithcode.com/dataset/brats-2017-1
IXI (1)	Information eXtraction from Images	http://brain-development.org/ixi-dataset/

RIRE (1)		http://insight-journal.org/rire
OASIS-3(1)	Open Access Series of Imaging Studies	https://www.oasis-brains.org/
**Liver**		
ADHD (1)	Massachusetts General Hospital	http://fcon_1000.projects.nitrc.org/indi/adhd200/
LiTS (3)	Liver tumor segmentation challenge	https://competitions.codalab.org/competitions/15595
CHAOS 1(2)	Combined (CT-MR) Healthy Abdominal Organ Segmentation	https://chaos.grand-challenge.org
MSD (1)	medical segmentation decathlon	https://decathlon-10.grand-challenge.org
SLIVER (1)	Segmentation of the Liver Competition	https://sliver07.grand-challenge.org/
**Lung**		
NIH ChestXray14 (3)	Radiologist-Level Pneumonia Detection on Chest X-Rays with Deep Learning	https://nihcc.app.box.com/v/ChestXray-NIHCC
